# Advancements in PARP1 Targeted Nuclear Imaging and Theranostic Probes

**DOI:** 10.3390/jcm9072130

**Published:** 2020-07-06

**Authors:** Ramya Ambur Sankaranarayanan, Susanne Kossatz, Wolfgang Weber, Mohsen Beheshti, Agnieszka Morgenroth, Felix M. Mottaghy

**Affiliations:** 1 Department of Nuclear Medicine, University Hospital Aachen, RWTH Aachen University, 52074 Aachen, Germany; rambursankar@ukaachen.de (R.A.S.); mbeheshti@ukaachen.de (M.B.); amorgenroth@ukaachen.de (A.M.); 2 Department of Nuclear Medicine, University Hospital Klinikum Rechts der Isar, Technical University Munich, 81675 Munich, Germany; s.kossatz@tum.de (S.K.); w.weber@tum.de (W.W.); 3 Central Institute for Translational Cancer Research (TranslaTUM), Technical University of Munich, 81675 Munich, Germany; 4 Department of Chemistry, Technical University of Munich, 85748 Munich, Germany; 5 Department of Nuclear Medicine and Endocrinology, Paracelsus Medical University, 5020 Salzburg, Austria; 6 Department of Radiology and Nuclear Medicine, Maastricht University Medical Center (MUMC+), 6202 Maastricht, The Netherlands

**Keywords:** PARP inhibition, PARP1 tracers, PARP1 theranostic probes, PET/SPECT imaging, Auger and Alpha emitters

## Abstract

The central paradigm of novel therapeutic approaches in cancer therapy is identifying and targeting molecular biomarkers. One such target is the nuclear DNA repair enzyme Poly-(ADP ribose) polymerase 1 (PARP1). Sensitivity to PARP inhibition in certain cancers such as gBRCA^mut^ breast and ovarian cancers has led to its exploitation as a target. The overexpression of PARP1 in several types of cancer further evoked interest in its use as an imaging target. While PARP1-targeted inhibitors have fast developed and approved in this past decade, determination of PARP1 expression might help to predict the response to PARP inhibitor treatment. This has the potential of improving prognosis and moving towards tailored therapy options and/or dosages. This review summarizes the recent pre-clinical advancements in imaging and theranostic PARP1 targeted tracers. To assess PARP1 levels, several imaging probes with fluorescent or beta/gamma emitting radionuclides have been proposed and three have advanced to ongoing clinical evaluation. Apart from its diagnostic value in detection of primary tumors as well as metastases, this shall also help in delivering therapeutic radionuclides to PARP1 overexpressing tumors. Henceforth nuclear medicine has now advanced towards conjugating theranostic radionuclides to PARP1 inhibitors. This paves the way for a future of PARP1-targeted theranostics and personalized therapy.

## 1. Introduction

DNA damage is recognized and repaired specifically by different repair mechanisms [1]. One of the early sensors of DNA single strand breaks is Poly ADP-Ribose Polymerase 1 (PARP1), a nuclear protein. PARP1 and other 16 PARP family members function as a catalyst for Poly (ADP-Ribosylation) (PARylation) using Nicotinamide Adenine Dinucleotide (NAD+) as the ADP donor [2]. PARP1 recognizes strand breaks, binds to the DNA backbone, recruits acceptor proteins, post-translationally modifies them by transferring PAR polymers (PAR-ylation) and also undergoes auto-PAR-ylation [2,3].Importantly, in cases of defective double strand DNA damage repair mechanisms (homologous recombination), possibly due to mutated Breast Cancer 1/2 (BRCA1/2^mut^) proteins, PARP1-mediated processes can take over the repair [2,4]. Unlike healthy cells, rapidly proliferating cancer cells are under higher replicative stress, which leads to genomic instability causing PARP1 overexpression. Hence, PARP1 is a critical protein, which has become an important target for inhibition therapies, especially in BRCA1/2^mut^ patients. This scenario, where simultaneous loss-of-function/inhibition of two complementary proteins resulting in cytotoxicity, is termed “Synthetic Lethality”. Till now, various PARP inhibitors (PARPis) such as olaparib (2014) [5], rucaparib (2016) [6], niraparib (2017) [7] and talazoparib (2018) have been clinically approved by the Food and Drug Administration (FDA) and European Medicine Agency (EMA) [8,9]. The additional use of DNA damaging agents might lead to an increased dependence on PARP1 activity for repair and by this would amplify a cells/tumors sensitivity to PARP inhibition. Combination therapies of PARPi with other DNA damaging therapies such as radiation therapy, chemotherapeutic drugs (e.g., Doxorubicin) [10] or anti-angiogenic therapy and immunotherapy are being assessed to improve cytotoxicity, and by this, the therapy efficacy and outcome as discussed in a recent review [11].

**Figure 1:**
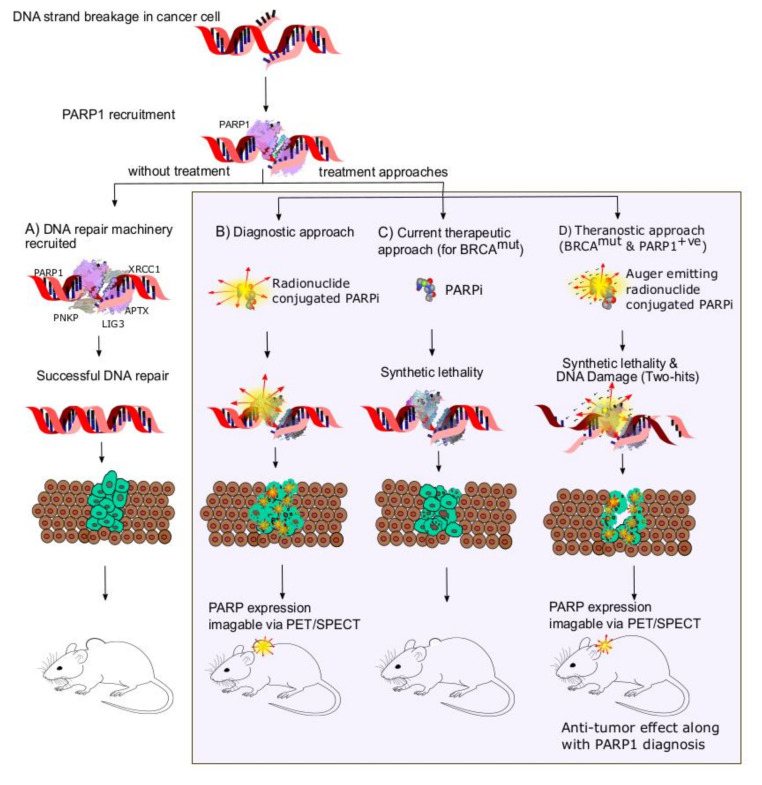
Schematic representation of PARP1-targeted therapy and imaging approaches. (**A**) Upon no treatment, PARP1-mediated repair enables cancer cell proliferation and tumour growth. (**B**) PET/SPECT radionuclide-conjugated PARP inhibitors enable imaging of the tumour tissues and PARP1-expression levels. (**C**) PARP inhibitor-based targeted therapy causes "Synthetic lethality", thereby inhibiting DNA repair mechanisms. (**D**) Auger electron (displayed in picture), Beta or Alpha particle-emitting radionuclides are able to cause DNA damage apart from synthetic lethality, and functions as a "two-hit" strategy and enhances apoptosis of cancer cells. For optimal synthetic lethality, supplementation with PARP inhibitors would be required.

With high prominence of PARPi in cancer therapy, determining PARP1 expression levels in tumors might help to predict the sensitivity to PARP1-targeted therapy. PARP-imaging agents are potentially useful in the pre-treatment phase as a guidance to predict therapy response and to facilitate patient stratification, and in interim and post-treatment phases to quantify tumor response to therapy. Initially, fluorescent tagged olaparib derivatives were developed for optical imaging, one of which (PARPi-FL) has progressed to a clinical trial for oral cancer detection upon topical application. Report on the first in-human trial shows quantifiable PARPi-FL-based tumor detection in human tissue specimens and feasible application methods for esophageal tumor imaging upon topical application of PARPi-FL [12,13]. The need for depiction of PARP1 expression on the whole-body level initiated design and development of radiolabeled PARPi derivatives, which led to non-invasive determination of PARP1 expression by imaging modalities like Positron Emission Tomography (PET) or Single Photon Emission Computed Tomography (SPECT). Synthesis of radiolabeled PARP1-targeting imaging probes have been in an accelerated drive in this past decade. Radiohalogens such as ^18^F, ^123^I, and ^131^I are favored for radiolabeling PARPis rather than radiometals (^68^Ga, ^99m^Tc) due to their ease of radiolabeling without the need of a chelator, apart from their favorable physical characteristics [14]. Nevertheless, there is a need to summarize and identify the advantages to determine the most relevant application and suitable candidates for clinical application. Hence, the purpose of this review is to compile and update on important PARP1-targeted radio-theranostics developed recently, in the context of their specific applications in cancer diagnosis and therapy. Figure 1 illustrates the different approaches in PARP mediated therapeutic, diagnostic and theranostics.

## 2. Pharmacokinetic Considerations

Clinically approved PARPi core structures or their derivatives have been used for labelling with radionuclides (Table 1). Ideally, pharmacokinetic defining parameters like the parent drug’s molecular weight (MW), charge, serum stability, vascular retention (% plasma protein binding/%PPB), lipophilicity (Log P_oct_ or Log P_CHI_ values), affinity (IC_50_), and PARP1 specificity should remain largely unchanged. These parameters will influence the in vivo behaviour such as tumor uptake and target to background ratios (TBR). Most reported radiotracers have shown an increase in lipophilicity and a predominantly hepatobiliary clearance. For example, the radionuclide conjugation of olaparib elevated lipophilicity for olaparib derivatives ^18^F-20 and ^18^F-PARPi from Log P_oct_ = 1.95 to Log P_oct_ = 2.51 and Log P_CHI_ = 2.15, respectively [15,16].

Due to the predominant nuclear target localization, PARPis usually diffuse passively across the plasma and nuclear membranes. For optimal passage across lipid bilayers, lipophilicity of Log P: 1.5–3.0 is optimal [31]. A further increase in lipophilicity (Log P_oct_ > 3.0) decreases passive diffusion across biological membranes, leading to low signal-to-noise ratios and hence is disadvantageous. Together with this, low dissociation constants (K_i_ = 1.2nM−5nM) prevent passive diffusion of PARPis out of the nucleus, avoiding quick wash-out [32]. Similarly, high %PPB (> 95%) reduces the tissue penetration ability, and thus decreases the drug uptake by organs, and would require a higher dosage application [15]. In the case of brain malignancies like glioblastoma multiforme (GBM), the penetration of the blood–brain barrier (BBB) is essential for imaging and therapy. For this, the optimal drug parameters are Log P_oct_:2–3, MW < 450 Da, and PPB < 95% [15]. PARPis have shown different BBB penetration abilities in preclinical studies. While talazoparib, olaparib, and rucaparib show limited penetration across intact BBB as they are liable to efflux by the BBB, veliparib and niraparib were shown to have better penetration [33,34]. However, results from a Phase I clinical trial (OPARATIC trial) showed that olaparib was able to accumulate in marginal and core tumors in GBM patients who were treated with low doses of temozolomide [35].

Tracers with favorable in vitro characteristics are then investigated for in vivo biodistribution. In vivo, blood half-life, stability, tumor targeting, and PARP1 specificity are essential parameters to understand the tracer behavior, its suitability to help delineate tumor vs. non-tumor tissue, and to identify targeting efficiency. It should be taken into consideration that biologically defined in vivo parameters like vascular permeability, tumor microenvironment, and cellular composition further impact the drug concentration and by this the efficacy of PARP-targeted therapeutics.

Taken together, all these parameters play a critical role to define the properties of a diagnostic or theranostic agent at the cellular as well as the systemic level (i.e., drug delivery to tumor tissue). Hence, optimal in vivo pharmacokinetics and related parameters are essential for clinical translation of PARP theranostics.

## 3. Imaging with PARP-Addressing Tracers

### 3.1. Radio-fluorinated PARP Tracers

The PET radionuclide ^18^F (half-life/T_1/2_ = 109.771 min) is one of the most favored diagnostic radionuclides in PARP imaging probes. ^18^F conjugated PARPis like olaparib (^18^F-Olaparib) [17], olaparib derivatives (^18^F-20 [15], ^18^F-PARPi [16]), and rucaparib derivatives (^18^F-Fluorthanatrace (^18^F-FTT) [25], ^18^F-WC-DZ-F [26]) have been evaluated for imaging of PARP1 expression.

The most recently published PARP1 tracer is an ^18^F-radiolabeled direct analog of olaparib (^18^F-Olaparib) that has caught significant attention [17]. Even though a low radiochemical activity yield of 18% ± 3% may be discouraging, its identical chemical composition to olaparib distinguishes it from other diagnostic tracers that are under investigation. Since it has similar pharmacokinetic and pharmacodynamic properties as its parental drug, apart from its use as a PARP1 targeting tracer, it can also provide insights into the systemic behavior of olaparib with regard to tumor accumulation and therapeutic dosage. Initial in vitro characterization using blocking studies in PSN-1, MiaPaCa-2, and Capan-1 cell lines showed in vitro PARP1 specificity. In a subcutaneous pancreatic ductal adenocarcinoma model, in vivo tumor uptake was enhanced upon radiation, confirming radiation-induced PARP1 overexpression. Recently Reilly et al., developed ^18^F-9e derived from the olaparib derivative AZD2461, for PARP1 imaging in neurodegenerative diseases. However, in spite of high PARP1 affinity (IC_50_ = 3.9 ± 1.2 nM, Log P = 2.26), the tracer was seen to be impenetrable across the BBB both in rodents and in primate models, which suggests the lack of BBB penetration ability of the parental drug [21].

Previously, ^18^F-conjugated tracers were developed as olaparib derivatives (^18^F-PARPi and ^18^F-20) and characterized in GBM models. Both tracers have similar structures: ^18^F-PARPi and ^18^F-20 have fluorobenzamide and methylfluorobenzamide moieties respectively in place of the isopropyl moiety of olaparib [15,16]. Recently, a simplified process for the synthesis of ^18^F-PARPi has been reported, reducing the synthesis time from 90 min to 66 min, although the radiochemical yield obtained was 9.6% compared to 10% reported earlier [36]. Both ^18^F-PARPi and ^18^F-20 have been shown in subcutaneous models promising tumor-to-muscle ratios of 5.1 ± 0.9 and 3.6 ± 0.5, respectively. While ^18^F-PARPi showed an encouraging tumor-to-brain ratio of 54.9 ± 14.1 (orthotopic model). ^18^F-20 suffered from heavy defluorination (>8.5%ID/g bone uptake, 1 h p.i), preventing it from further investigation [16]. In further preclinical studies, ^18^F-PARPi was used to quantify target engagement of clinical PARPis (olaparib and talazoparib) in Non-Small Cell Lung Cancer (NSCLC) models upon co-treatment. This study enabled deciphering dosage regimens for complete drug–target engagement (olaparib: 15 mg/kg; talazoparib—5 mg/kg) and tumor residence times (half-lives: olaparib—9.4 h; talazoparib—9.8 h) [37]. It was also used to monitor the target engagement of talazoparib where therapeutic and subtherapeutic dosage was distinguishable by differences in ^18^F-PARPi uptake [38]. ^18^F-PARPi was also studied as an alternative to ^18^F-FDG for the delineation of oral cancer tissue from surrounding healthy tissue [39]. Furthermore, ^18^F-PARPi has also proved to better differentiate radiation necrosis from tumors compared to ^18^F-FET (in GBM models), and malignant from inflamed lymph nodes in B-cell lymphoma models [40,41].

Apart from olaparib derivatives, a rucaparib derivative, ^18^F-WC-DZ-F has been characterized in a subcutaneous prostate cancer model [26]. Structurally, it is an analogue of ^18^F-FTT, which is currently in clinical trials. ^18^F-WC-DZ-F was developed by replacing the ^125^I in ^125^I-KX1 with ^18^F, in order to improve the pharmacokinetics, in vivo tumor uptake and enhance blood stability. The tumor uptake was close to 4% ID/c.c. (2 h p.i) as detected by PET imaging. However, ex vivo biodistribution data from naive mice showed unspecific uptake in tissues such as bone and muscle. Additionally, since TBRs and correlation with PARP1 expression levels were not reported, further studies will be needed to validate this tracer.

Other olaparib-derived ^18^F- tracers (^18^F-FTT, ^18^F-BO and the dual modality PET/fluorescent imaging agent ^18^F-PARPi-FL) have also been investigated. As they were already reviewed elaborately by previous reviews, they are spared from detailed discussion in this article to avoid redundancy [4,20,22,42]. Briefly, ^18^F-BO has been tested in ovarian, breast, and pancreatic cancer models where uptake correlated with PARP1 expression. ^18^F-PARPi-FL showed higher specificity. However, it showed heavy in vivo defluorination (> 10% ID/g bone uptake). ^18^F-FTT was first validated in a subcutaneous breast cancer model showing promising tumor uptake (4% ID/cc, 1 h p.i) [25]. It was also successfully validated in vitro and in vivo breast cancer models to image PARP1 expression levels and has progressed to clinical trials [43].

Besides PARPi-derived tracers, a substrate-based tracer (^18^F-labelled NAD analog), named ^18^F – Substrate-based PARP Activity Radiotracer (^18^F-SuPAR) has been developed for imaging PARP-1/2 activity. The N_6_ of the adenine moiety in NAD is substituted with fluorinated poly-ethylene glycol (F-PEG_2_) prosthetic groups. Blocking experiments showed significant uptake reduction in an orthotopic breast cancer model but not in a subcutaneous model. However, correlation of ^18^F-SuPAR accumulation and PAR levels in tumor sections proved the tracer specificity. PET images post external beam irradiation showed an increase in tumor uptake. The major disadvantage is that, although the modification of NAD^+^ is optimized for PARP1/2 uptake, NAD^+^ is not a PARP1-specific substrate, as seen in vitro and in vivo by the background uptake possibly by other oxidoreductase enzymes. Moreover, rapid clearance and low serum stability (T_1/2_ < 60 min) are other hindrances for its use as a PARP1 imaging agent [30].

While the number of investigations on synthesizing and optimizing new PARP1 tracers are fast growing, two tracers, ^18^F-PARPi and ^18^F-FTT, have now progressed to Phase I clinical trials.

There are currently two Phase I clinical trials related to ^18^F-PARPi. In a head and neck cancer imaging trial (NCT03631017), ^18^F-PARPi administration was safe and well tolerated. It was shown that all ^18^F-FDG avid lesions also showed ^18^F-PARPi uptake with comparable contrast ratios. Interestingly, several lymph nodes that were ^18^F- PARPi, but not ^18^F-FDG avid, resolved after chemoradiation [44]. In a second, ongoing clinical trial, ^18^F-PARPi is investigated for imaging of brain tumors (NCT04173104).

**Figure 2:**
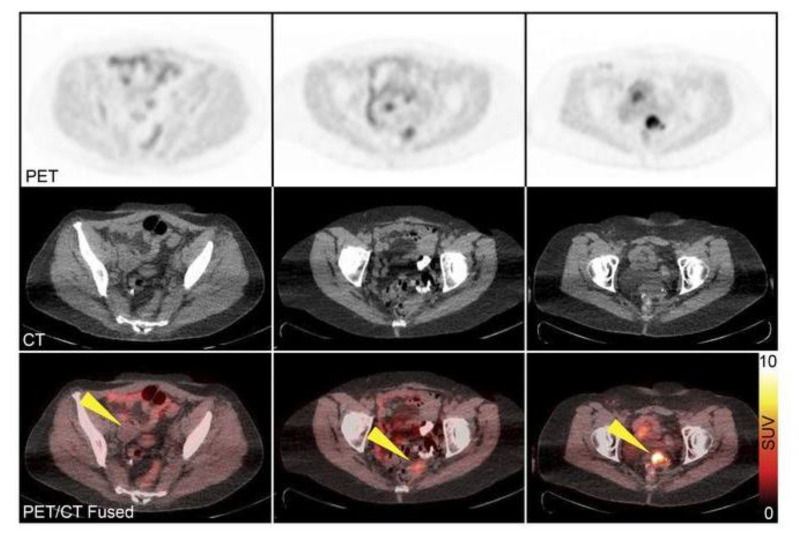
PET/CT images of ^18^F-FTT uptake in ovarian cancer patients. PET/CT images from a clinical trial (NCT02637934) in three ovarian cancer patients show a wide range of ^18^F-FTT uptake in tumor lesions. Standard uptake value (SUV) ranges from 2 (top-left) to 12 (top-right). Yellow arrows indicate sites of tumor. Reproduced with permission from Makvandi et al., titled “A PET imaging agent to evaluate PARP expression in ovarian cancer”, published by The Journal of Clinical Investigation, 2018 [46].

The other PARP1 tracer undergoing clinical evaluation is ^18^F-FTT. Several trials are ongoing, which are listed in ClinicalTrials.org to study ^18^F-FTT as a PARP1 tracer pre-/post-treatment, in a wide range of cancers like ovarian (NCT03604315, NCT02637934), breast (NCT03846167), pancreatic (NCT03492164), prostate (NCT03334500), and GBM patients (NCT04221061). The first in-human trials showed promising uptake by tumor tissue in a cholangiocarcinoma patient [45]. Recently, a Phase I trial report using ^18^F-FTT in a cohort of ovarian cancer patients pre-treated with chemotherapy showed discernible tumor uptake, inter-tumor heterogeneity, and positive correlation between high ^18^F-FTT uptake and platinum-treatment resistance (Figure 2). Moreover, the study reports no correlation between uptake of ^18^F-FDG and ^18^F-FTT. ^18^F-FDG and ^18^F-FTT were shown to give complementary information enabling detection of metastatic omental lesions. Immunohistochemistry of clinical specimens showed PARP1 overexpression in lymph nodes with and without nodal disease. As a result, an accurate differentiation between malignant and reactive/inflammatory lymph nodes was not possible. Even though the study mentions high ^18^F-FDG and low ^18^F-FTT uptake in one patient having inflammatory lymph nodes, further investigation is required with a larger cohort [46].

### 3.2. Radioiodinated PARP1 Tracers

PARP inhibitors labeled with different PET/SPECT radioisotopes of iodine (^123/124/125/131^I), have also been developed initially as imaging tracers and furthermore, their theranostic efficacy was explored. Since Iodine has a larger molecular weight, the pharmacokinetics of radio-iodinated tracers vary greatly from the parent drug. In 2015, two independent studies reported the synthesis of iodinated olaparib derivatives. Both studies characterized the same tracer backbone structure using different synthesis protocols and labelled with different radioisotopes of iodine (^123/125^I and ^131/124^I2-PARPi) [18,19].

In the report by Salinas et al., ^131/124^I2-PARPi (T_1/2_ = 8.01 d for ^131^I; 4.17 d for ^124^I) was characterized and optimized as a potential PET/SPECT tracer in GBM models. Biodistribution at 2.5 h post i.v injection showed a remarkable tumor-to-brain ratio of 40.0 ± 6.3, and a tumor-to-muscle ratio of 13.7 ± 4.1, confirming tumor targeting and retention. Unexpectedly, in their biodistribution studies with a subcutaneous model, there was no direct correlation between an increase in specific activity of the administered tracer with an increase in tumor-to-muscle ratio. But heavy deiodination was seen, as the tumor-to-thyroid ratio was 1.82 ± 0.25, in spite of prior thyroid blocking with sodium iodide (NaI). This is possibly due to the tracer oxidation in the liver. Notably, tumor uptake at 2 h in a U87 subcutaneous model was reported as 0.17% ID/g (tumor: muscle = ~4.36) whereas in the U251 orthotopic model it was 0.43% ID/g (tumor: muscle = 13.7 ± 4.1) despite the same PARP1 expression levels in both U87 and U251 tissues. This can be explained by a possible disruption in the BBB of the orthotopic model, improving passive targeting to the brain [18].

Similarly, Zmuda et al. reported conjugation of SPECT radionuclides ^123/125^I, (T_1/2_ = 13.22 h for ^123^I; 59.49 d for ^125^I) to the same precursor and similar coupling conditions as Salinas et al. The biodistribution in a subcutaneous GBM model showed a TBR similar to that of the earlier reported tracer. Even though high plasma protein binding (96.2%) is tolerable for imaging of primary tumor due to BBB disruptions, it is not optimal for PARP1 imaging of metastasis with an intact BBB [19].

These two studies were the first reports on radioiodine tagging as a convincing imaging strategy for PARP1 expression.

In 2016, two studies reported the synthesis and biodistribution of radio-iodinated benzimidazole PARPi (AG14032) derivatives (^125^I-KX-02-019 and ^125^I-KX1), analogous of ^18^F-FTT [27,28]. Though ^125^I-KX1 showed high tumor uptake (~5% ID/g at 2 h p.i), olaparib pre-injection (i.p.) did not reduce tracer accumulation in the tumor, which is the standard way to evaluate PARP specificity. Furthermore, biodistribution of ^125^I-KX-02-019 showed heavy deiodination (Thyroid uptake ~5% ID/g vs. tumor uptake ~1%ID/g) at 4 h p.i.

### 3.3. PARP1 Targeted Theranostics

Most recently, theranostic PARP radio-ligands have been developed. Here, therapeutic radionuclides, i.e., α, β^-^, and auger emitters, were conjugated to PARPis with the goal of effectively inflicting DNA damage on cancer cells, as binding to PARP1 leads to radioactive decay events in close proximity of the DNA. Importantly, these radionuclides also emit either positrons or γ-rays and hence allow evaluation of the tracer’s in vivo behavior via PET/SPECT imaging. For theranostic PARP tracers, their therapeutic effect is not mitigated by PARP inhibition, but the PARP inhibitor acts as a delivery vehicle for the cytotoxic radiation. Theranostic radionuclides were either chosen for their short range, high linear energy transfer (LET) alpha (α) particle emissions (LET: 80–100 keV/μm upto 100μm), auger electron emissions (LET 4–26 keV/µm, upto ~0.5 μm), or long range, low LET beta (β) emissions (LET ~0.2 keV/µm, upto 1 cm) [47].

#### 3.3.1. α- emitter Theranostics

Preclinical studies have been reported in neuroblastoma models with ^211^At-MM4, a rucaparib derivative (KX1) conjugated to an α- particle-emitting SPECT tracer ^211^At (T_1/2_ = 7.5 h). Ex vivo biodistribution showed a rapid renal clearance, but tumor uptake increased from ~1% ID/g at 2 min p.i to 14% ID/g at 2 h p.i. upon intravenous administration, with a promising tumor-to-blood ratio of 7.5. Therapeutic efficacy in mice showed a remarkable increase in median survival from 35 d (untreated) to 61 d (single dose) and even further to 80 d (multiple fractionated doses). These results are promising, especially as the animals showed no weight loss, no tumor regrowth, and minimal residual tumor at the end of 80 d, indicating low systemic toxicity at efficacious doses [29].

#### 3.3.2. β- emitter Theranostics

The radionuclide ^64^Cu (T_1/2_ = 12.7 h), which emits β^-^ particles for therapy and positrons for PET imaging was conjugated to DOTA-PARPi, derived from olaparib [23]. This tracer was characterized in mesothelioma models, with biodistribution showing peak tumor uptake at 1 h p.i (3.45 ± 0.47%ID/g), however tumor retention was poor as the tumor-to-muscle ratio at 18 h was ~1. Moreover, the conjugation of DOTA moiety reduced the binding affinity and thereby the cytotoxicity by 40 times. This would require an increase in the therapeutic dosage, which will in-turn increase systemic toxicity, and consequently limit its theranostic ability [23].

The earlier described ^131^I-PARPi can also be used as a theranostic compound, since ^131^I is a γ and β^-^ emitter. Jannetti et al. investigated its therapeutic efficacy in a subcutaneous GBM model [48]. Intratumorally administered fractionated doses (3x of 14.8 MBq) over 6d slowed tumor growth, increasing median survival from 20d (“cold” ^127^I-PARPi treated) to 29 d (^131^I-PARPi treated). Two weeks after the last dose, tumor growth progression was observed and showed a linear growth rate similar to that of control (PBS) and “cold” ^127^I-PARPi-treated cohorts. This can be explained by limited on-target residence time and/or the half-life (T_1/2_ = 8 d) of ^131^I. To mimic a Convection Enhanced Delivery (CED), an osmotic pump mediated delivery into brain was investigated. In this orthotopic model, feasibility of strongly increasing brain uptake in tumor mice compared to naïve mice was shown, although the tumor vs. healthy brain tissue uptakes (tumor-to-brain ratio) was not reported. Although intratumoral applications are used for targeted delivery into brain malignancies in clinical studies, tumor–brain delineation is critical to assess and avoid neurotoxicities. Therefore, careful precaution with dosage is needed in case of intratumoral application of ^131^I in glioblastoma to avoid any risk of bystander effect on surrounding healthy brain tissues [49,50].

#### 3.3.3. Auger Emitter Theranostics

Use of auger emitters (^123/125^I) in PARP theranostic agents was reported by Lee et al., with ^125^I-KX1. The cytotoxic efficiency in neuroblastoma cell lines treated with ^125^I-KX1 was 10^4^-10^6^ times higher than its non-radioactive precursor KX1. DNA damage induction was significantly higher compared to veliparib treatment as measured by pH2AX fluorescence intensity [51]. Its PARP1 specificity was demonstrated by Makvandi et al., where PARP1 KO ovarian cancer cell lines showed reduced ^125^I-KX1 uptake [46]. However, further in vivo survival studies are required to evaluate its anti-tumor efficiency.

Another auger-emitting theranostic tracer, ^123^I-MAPi (Iodine-123 Meitner-Auger PARP1 inhibitor), an isotopologue of ^131^I-PARPi, has been studied in GBM models [24]. Survival increased from 40d to 58d upon intratumoral delivery. Intratumoral delivery to an orthotopic model (using an osmotic pump) showed a further prolonged survival to 72d. Of note, intrathecal delivery has reported to result in stress-related deaths in mice, which was attributed to the small volume of the murine skull, and hence should only be a limiting factor in small animal studies. These results support the note that auger emission-mediated anti-tumor efficiency is promising.

Further studies are required for a comparison of the therapeutic efficacies of the auger emitting theranostic probes ^125^I-KX1 and ^123^I-MAPi.

In neuroblastoma 3D solid tumor models, comparison of the cytotoxic efficiencies of ^125^I-KX1 with other PARP1-targeted (^211^At-MM4) or non-PARP1-targeted (^125^I-MIBG) radiopharmaceuticals showed that ^125^I-KX1 was less effective compared to ^211^At-MM4 in terms of concentration, tumor dosage (350x lower per decay), and tumor-cell nuclei dosage (150x lower per decay) [51]. The theranostic ability of ^211^At is comparably advantageous to ^125^I due to its superior cytotoxicity, and thereby low dosage requirement in addition to its favorable physical half-life. Taken together, amongst the theranostic PARP tracers, ^211^At-MM4 has shown the most promising in vivo anti-tumor efficiency.

## 4. Future Prospects and Conclusion

PARP1 imaging is emerging as a novel tool for assessing PARP1 expression in tumors and monitoring PARP inhibitor therapy response in the clinic. The rising interest in this field is evident from the development of several tracers in a short time span. The PARP1 tracers discussed in this work have been evaluated in various cancer types, which all show PARP overexpression. In vivo tumor characterization facilitated by PARP1 imaging could become a valuable instrument for personalized therapy in patients, which is the need of the hour.

Among the tracers reported, ^18^F-FTT and ^18^F-PARPi have progressed to clinical trials showing promising results in ovarian cancer and head and neck cancer patients, respectively and are further investigated in several types of solid tumors. Upon reproducible validation in the future, ^18^F-Olaparib could also be a potential candidate for clinical trials due to its chemical identity to olaparib. Some hurdles that lay before translation of other tracers include improving serum stability, optimizing tumor uptake, tackling in vivo dehalogenation, and avoiding off-target uptake.

For theranostics, primary challenges include optimizations on a) tumor residence time vs. absorbed dose, b) tumor vs. clearance-organ uptake, c) compromise between T_1/2_ of the chosen radionuclide and its therapeutic efficiency. Among the reported theranostic compounds, the α emitting ^211^At-MM4 has shown encouraging anti-tumor effects. However, considerable cytotoxicity on clearance organs such as liver and stomach, as well as its susceptibility to de-astatination are persisting challenges. Moreover, patients pre-treated with platinum-based chemotherapy show elevated PARP1 levels in the tumor microenvironment. Though this can lead to an overestimation of tumor size by imaging tracers, the high PARP1 levels in the tumor microenvironment can enhance theranostic tracer uptake leading to better therapeutic efficacy.

Particularly for GBM, crossing the BBB is a roadblock for small molecule drugs including most PARP inhibitors. Feasibility of bypassing the BBB via intrathecal application or intratumoral delivery by CED was shown preclinically and could be an approach to efficiently deliver theranostic PARPi to brain malignancies [52]. Nevertheless, alternative minimally invasive strategies (e.g., intravenous injection) to address brain and other malignancies should also be developed to minimize the risk of infection, neural toxicity, pain, and patient discomfort. For non-invasive tumor-targeted delivery, nanomedicines could be a possible solution [53,54]. Recent preclinical studies in glioma models present nanoparticles developed using poly-MPC coating as an effective way to cross BBB [55,56]. Nano-formulations of PARPi have already been reported, although none with radiotracers. Nanoemulsion-based delivery of PARPi-FL showed increased blood half-life, and delineated subcutaneous xenografts of small cell lung cancer [57]. Liposomal talazoparib showed significant increase in survival and reduction in side effects [58]. Loading radiolabeled drugs in nanoparticles is challenging, but could overcome current limitations for PARP-targeted alpha and auger emitters, as this can potentially reduce off-target DNA damage by diminishing off-target uptake [59]. Nanoscale delivery systems have proven to minimize side-effects, for e.g., the in-use lysosomal doxorubicin (Doxil®) [60]. This calls for further research towards developing mechanisms for targeted delivery to tumors, which will improve their future prospects.

Taken together, PARP1 overexpression in various cancers can be exploited as a molecular target in the clinic. Imaging and therapy tracers have been developed with promising preclinical results and a number of relevant clinical applications have been outlined. Upon optimization, some tracers are fast approaching clinical translation.

## Figures and Tables

**Table 1:** Summary of the PARP tracers, their respective precursors, modality of imaging, and their current stage of development. Chemical structures of parent PARP inhibitors and their derivative radiotracers show structural modifications (green) and radionuclides (red). Modality of Imaging shows their PET (Positron Emission tomography)/SPECT (Single photon emission computed tomography) tracer capability.

Parent	Tracer	Modality of Imaging	Development Phase
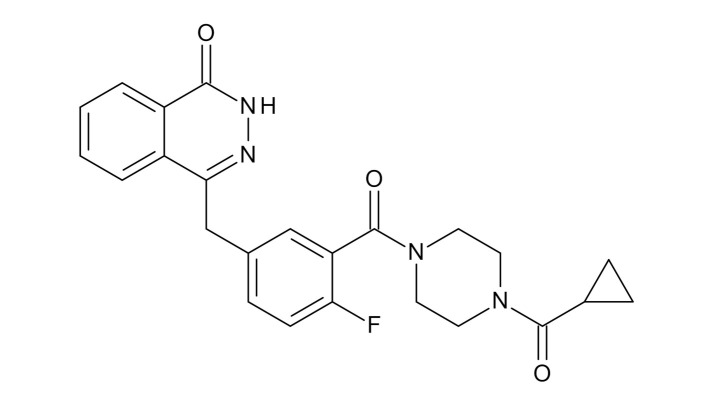 Olaparib	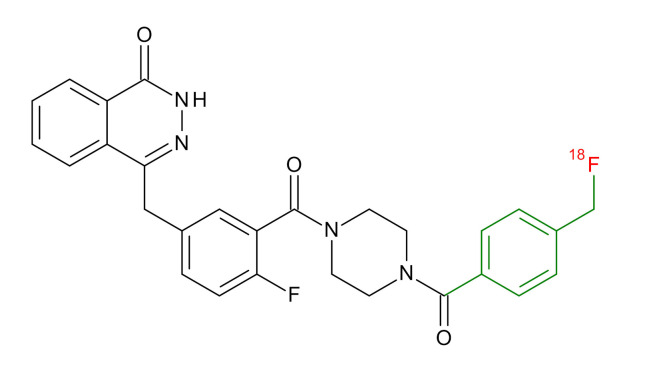 ^18^F-20 Zmuda et al., [15]	PET, Optical	Preclinical
	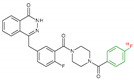 ^18^F-PARPi Carney et al., [16]	PET, Optical	Clinical Trials NCT03631017, NCT04173104
	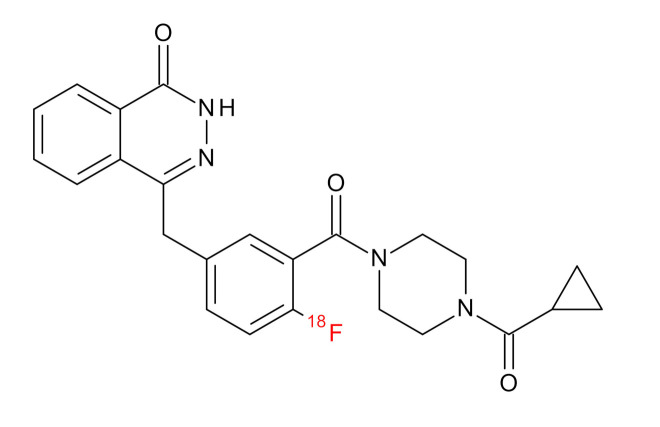 ^18^F-Olaparib Wilson et al., [17]	PET, Optical	Preclinical
	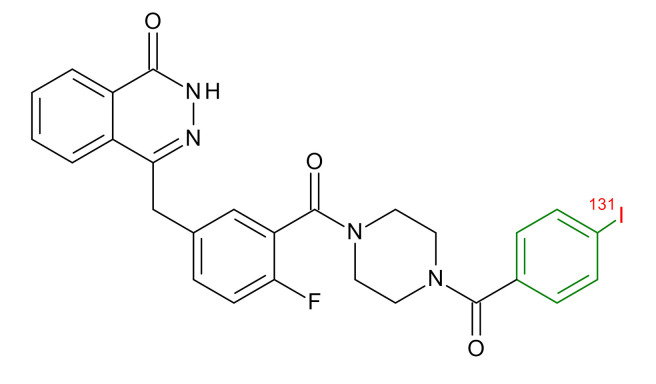 ^131^I-PARPi Salinas et al., [18]	PET, Therapy	Preclinical
	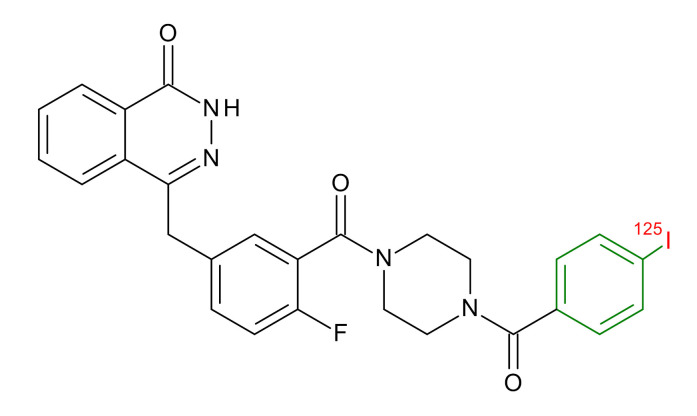 ^125^I-PARPi Zmuda et al., [19]	PET, Optical	Preclinical
	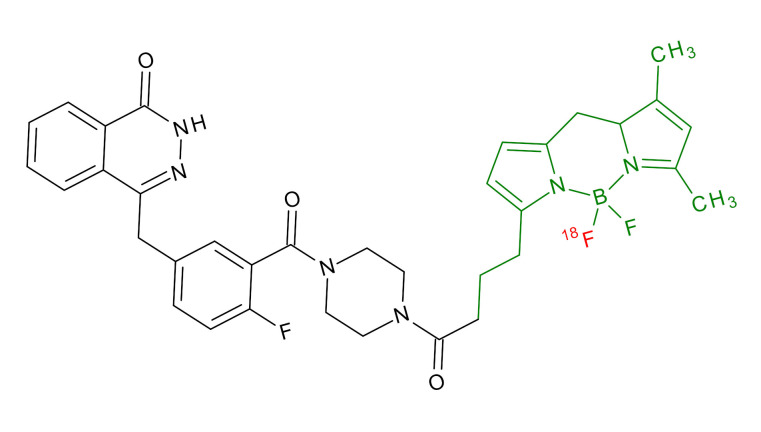 ^18^F-PARPi-FL Keliher et al., [20]	PET, Optical	Preclinical
	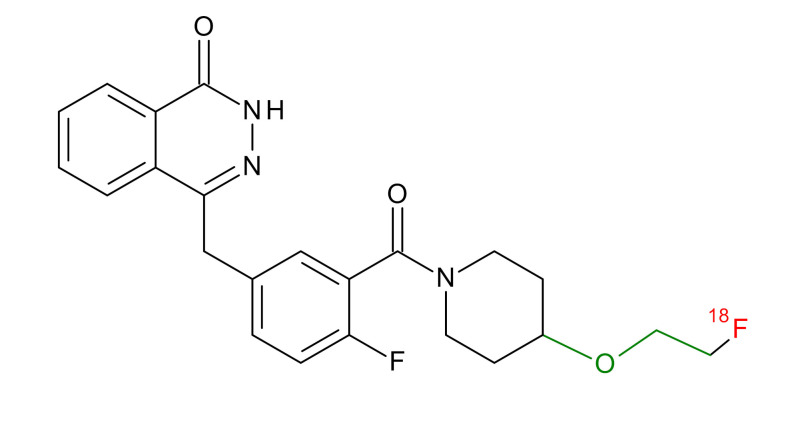 ^18^F-9e Reilly et al., [21]	PET, Optical	Preclinical
	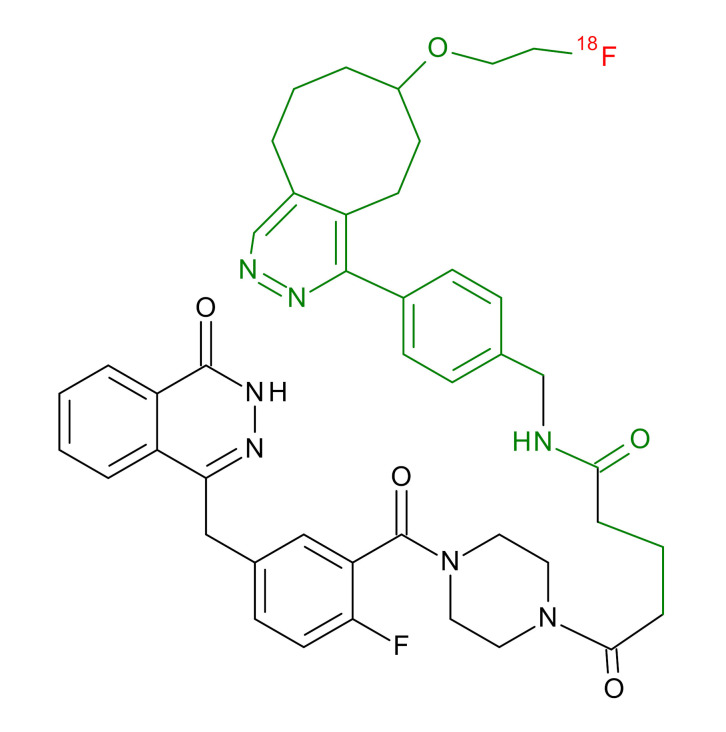 ^18^F-BO Reiner et al., [22]	PET, Optical	Preclinical
	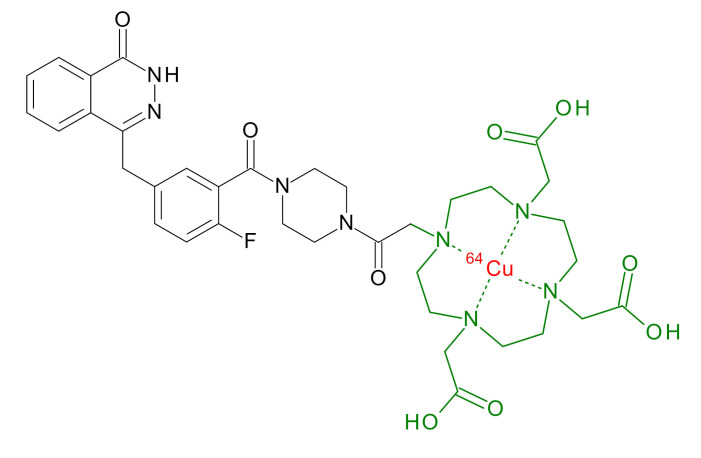 ^64^Cu-DOTA-PARPi Huang et al., [23]	PET, Therapy	Preclinical
	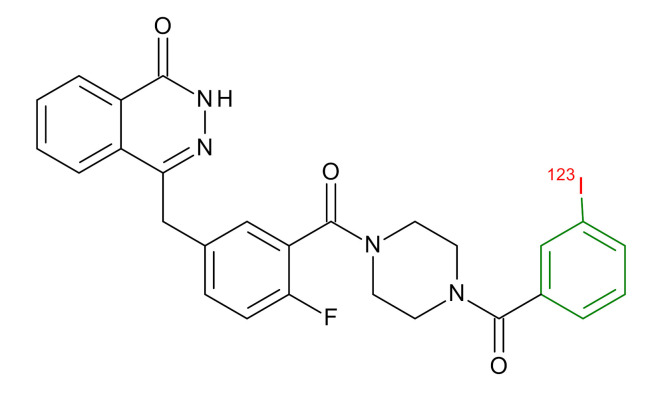 ^123^I-MAPI Pirovano et al., [24]	SPECT, Therapy	Preclinical
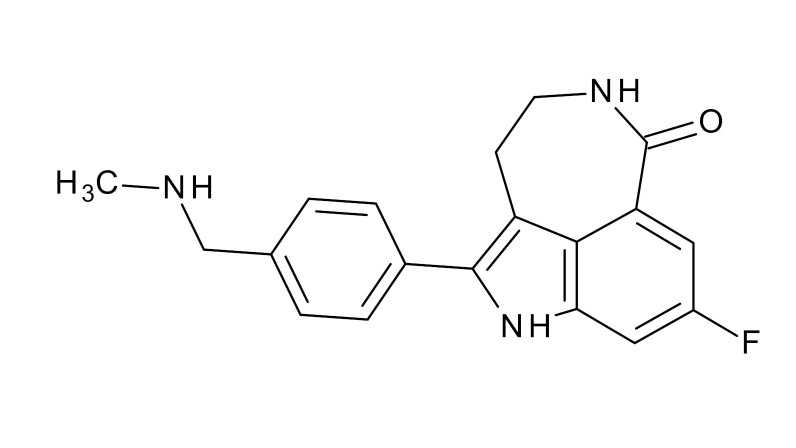 Rucaparib	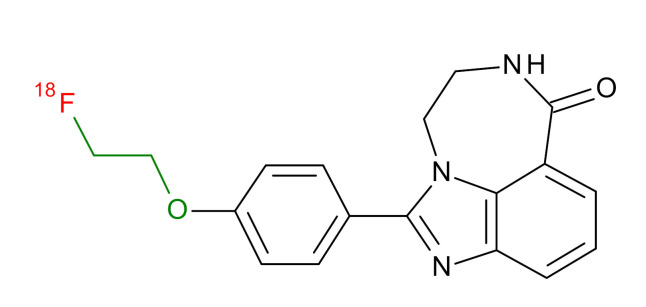 ^18^F-FTT Zhou et al., [25]	PET, Optical	Clinical Trials NCT03604315, NCT04221061, NCT03492164, NCT03846167, NCT03083288, NCT03334500, NCT02637934, NCT02469129.
	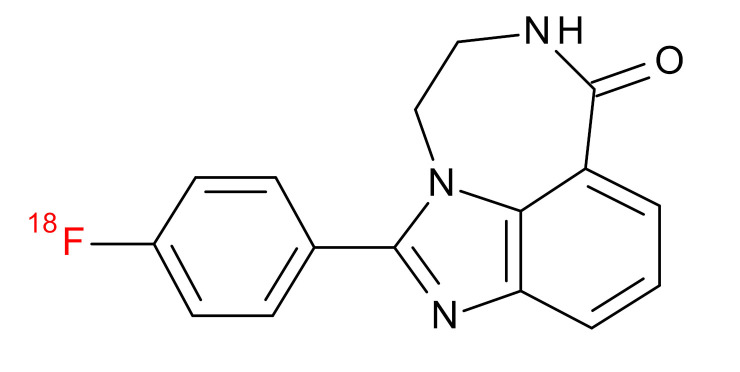 ^18^F-WC-DC-F Zhou et al., [26]	PET, Optical	Preclinical
	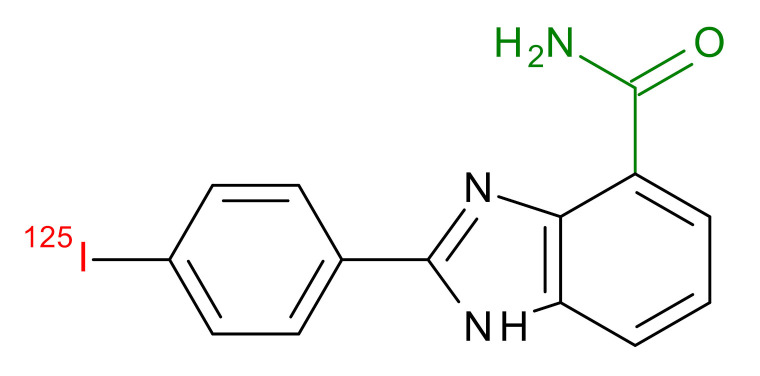 ^125^I-KX-02-019 Anderson et al., [27]	SPECT, Optical	Preclinical
	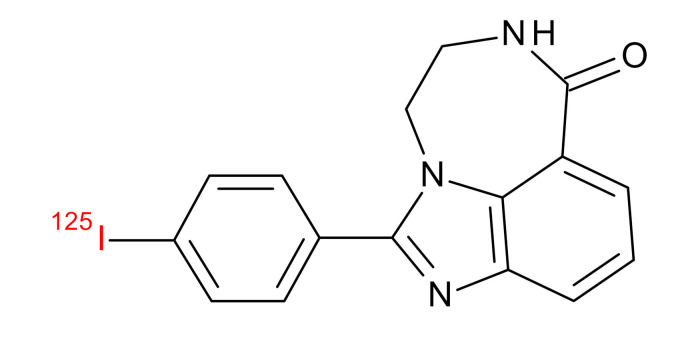 ^125^I-KX-1 Makvandi et al., [28]	SPECT, Therapy	Preclinical
	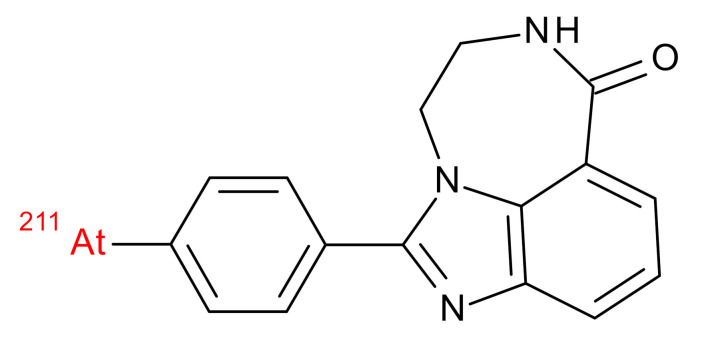 ^211^At-MM4 Makvandi et al., [29]	Therapy SPECT	Preclinical
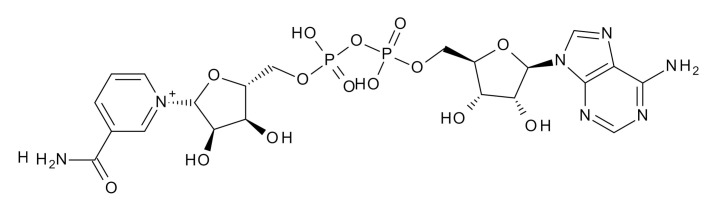 NAD+	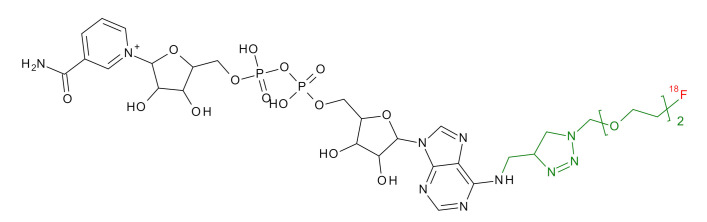 ^18^F-SUPAR Shuhendler et al., [30]	PET, Optical	Preclinical

## Abbreviations

PARP1	Poly ADP Ribose Polymerase 1
NAD	Nicotinamide Adenine Dinucleotide
PPB	Plasma protein binding
GBM	Glioblastoma multiforme
BBB	Blood–brain barrier
FDG	Fluorodeoxyglucose
DOTA	Dodecane Tetraacetic acid
